# Structure and properties of (Nd,Sr)(Al,Ta)O_3_ (NSAT) substrate crystals

**DOI:** 10.1107/S2052520626000703

**Published:** 2026-04-01

**Authors:** Roberts Blukis, Christian Rhode, Thilo Remmele, Elias Kluth, Rüdiger Goldhahn, Martin Feneberg, Ambra Celotto, Giorgio Divitini, Mario Brützam, Darrell G Schlom, Christo Guguschev

**Affiliations:** a Leibniz Institute for Crystal Growth (IKZ), Max-Born-Str. 2, 12489Berlin, Germany; b Institut für Physik Otto-von-Guericke-Universität Magdeburg Universitätsplatz 2 D-39106Magdeburg Germany; c Istituto Italiano di Tecnologia via Morego 30 16163 Genoa Italy; d Department of Materials Science and Engineering Cornell University Ithaca NY14853-1501 USA; e Kavli Institute at Cornell for Nanoscale Science, Ithaca, NY14853-1501, USA; University of Warwick, United Kingdom

**Keywords:** NSAT, single-crystal substrate, cubic perovskite, oxide single crystal, crystal growth

## Abstract

An emerging cubic substrate material (Nd,Sr)(Al,Ta)O_3_ (NSAT) has been analysed using powder and single-crystal X-ray diffraction, transmission electron microscopy, infrared (IR) and ultraviolet-visible range ellipsometry as well as IR- and Raman spectroscopies. Based on this experimental characterization, the structure and vibrational properties are described with improved understanding. On a large length scale the NSAT substrate is found to have a basic simple cubic perovskite structure (*a* = 3.8427 Å) that, on a small length scale, is subdivided into superstructure ordered nanoscale domains with doubled lattice (unit-cell) parameter (*a* = 7.6855 Å). Ellipsometry and vibrational spectroscopy show absorption bands consistent with the expected structure type, however, the peak widths indicate significant local deviations from the perfectly ordered superstructure or ideal solid solution. Since the lattice parameter is unaffected, the observed inhomogeneity is not expected to affect the use of NSAT as a substrate for most applications.

## Abstract 2

An emerging cubic substrate material (Nd,Sr)(Al,Ta)O_3_ (NSAT) has been structurally and spectroscopically characterized revealing that the nominally cubic NSAT consists of partially developed cubic superstructure domains with doubled unit-cell parameter. The spectroscopic features are consequently broad due to this partial order.

## 1. Introduction

Technological advances in thin film technology require increasingly high-quality substrates with very specific unit-cell (lattice) parameters. (Nd,Sr)(Al,Ta)O_3_ (NSAT) is a cubic *AB*O_3_ perovskite with the *A* site being occupied by either Nd or Sr and the *B* site by Al or Ta and has *Pm*3*m* symmetry to a reasonably good approximation (as described later in the text). In its most common form the chemical composition is Nd_0.4_Sr_0.6_Al_0.7_Ta_0.3_O_3_ and it has a corresponding lattice parameter of *a* = 3.8353 Å at 293 K (Ito *et al.*, 2002*b* ▸; Pawlak *et al.*, 2005 ▸). Due to this lattice parameter it is considered as a useful substrate material for the deposition of GaN (Ito *et al.*, 2002*b* ▸; Pawlak *et al.*, 2005 ▸; Shimamura *et al.*, 1998 ▸) and thin films of the cuprate superconductors (Mateika *et al.*, 1991 ▸). The phase diagram where NSAT resides is not known, however, NSAT is very similar to (La,Sr)(Al,Ta)O_3_ (LSAT) for which a pseudo-binary phase diagram is partially known. Specifically LSAT exists on the Sr(Al_0.5_Ta_0.5_)O_3_ (SAT) rich side from SAT to 50% SAT-50% LaAlO_3_, near melting temperatures (Berkowski *et al.*, 2003 ▸). It melts congruently at a composition of about (LaAlO_3_)_0.21_–(SrAl_0.5_Ta_0.5_O_3_)_0.79_ (Berkowski *et al.*, 2003 ▸), which is an indifferent point (the liquid–solid equivalent of an azeotrope) (Fratello *et al.*, 2024 ▸). By the same analogy, NSAT can be considered belonging to a pseudobinary phase system between SAT and NdAlO_3_, where the SAT-rich part of the phase diagram down to about 50 mol% SAT is a cubic solid solution, although the exact compositional limits and composition of the indifferent point of NSAT are unknown. As a solid solution, the lattice parameter of NSAT can be changed within some range by changing the Nd/Sr ratio or by replacing some Nd by La (Ito *et al.*, 2002*b* ▸).

NSAT has a smaller unit-cell parameter compared to LSAT, making it a particularly close match to high transition temperature (*T*_c_) cuprate superconductors like Bi_2_Sr_2_CaCu_2_O_8+δ_ with *T*_c_ = 89 K (Sunshine *et al.*, 1988 ▸; Yu *et al.*, 2019 ▸) and Bi_2_Sr_2_Ca_2_Cu_3_O_10+δ_ with *T*_c_ = 108 K (Watanabe *et al.*, 2003 ▸). NSAT is also well lattice matched to ferroelectrics, such as Bi_4_Ti_3_O_12_ (Uecker *et al.*, 2017 ▸; Martin & Schlom, 2012 ▸), the metal-insulator transition material Ca_2_RuO_4_ (Alexander *et al.*, 1999 ▸) as well as the highly conducting and optically transparent oxide SrVO_3_ (Zhang *et al.*, 2016 ▸; Lan *et al.*, 2003 ▸; Moyer *et al.*, 2013 ▸).

In contrast to LSAT, which is well known, available commercially, and used extensively as a substrate, NSAT is fairly poorly characterized and crack-free crystals, suitable for the preparation of large substrate samples, have not been demonstrated so far (Ito *et al.*, 2002*b* ▸). There is even some controversy surrounding the structure of NSAT. It has been proposed to belong to a *Pm*3*m* cubic (Ito *et al.*, 2002*b* ▸; Mateika *et al.*, 1991 ▸) or *Fm*3*m* cubic (this study) space group. By analogy with LSAT, to which NSAT is very similar, it could also be proposed to belong to *Pn*3*m* cubic (Pawlak *et al.*, 2005 ▸), *Fm*3*m* cubic (Li *et al.*, 2003 ▸) or *I*4 tetragonal (Steins *et al.*, 1997 ▸) space groups. All studies agree on the basic perovskite structure type but disagree on potential distortions and the description of the superstructure ordering. LSAT has been proposed to consist of a nanoscale mixture of superstructure ordered and disordered domains, with a high likelihood of NSAT displaying the same or similar features (Li *et al.*, 2003 ▸). Such features could impact the performance of NSAT substrates and hence are worthy of investigation. Other physical characteristics of NSAT, such as dielectric and optical properties have also not been reported. Here we have used a combination of X-ray scattering techniques [powder and single-crystal X-ray diffraction (XRD)] and transmission electron microscopy (TEM) methods (scanning transmission electron microscopy (STEM) with high-angle annular dark field (HAADF) imaging and STEM energy dispersive spectroscopy (EDS)] to elucidate the structural behaviour of NSAT in significantly greater detail than has been reported before. The diffraction and imaging methods are supplemented by infrared (IR) and ultraviolet-visible (UV–vis) range ellipsometry as well as IR and Raman spectroscopy.

## 2. Material and methods

### 2.1. NSAT single-crystal growth

The NSAT samples analysed in this study were grown using a Czochralski method from melt of composition Nd_0.396_Sr_0.604_Al_0.698_Ta_0.302_O_3_ or equivalently (NdAlO_3_)_0.396_–(SrAl_0.5_Ta_0.5_O_3_)_0.604_. The starting material was prepared by mixing dried powders of Nd_2_O_3_, SrCO_3_, Al_2_O_3_ and Ta_2_O_5_ of 4N purity (except SrCO_3_ which had 5N purity) followed by calcination at 1473 K for 15 h. The powder mixture was then pressed into cylindrical bars by cold isostatic pressing at 2000 bar in order to optimize the crucible filling process. The crystal growth experiments were performed using a conventional RF-heated Czochralski setup equipped with a crystal balance. During growth automatic diameter control was enabled and an atmosphere of 99.999% pure nitrogen at 1 bar pressure was employed. Iridium crucibles (38 mm inner diameter) embedded in ZrO_2_ and Al_2_O_3_ insulation were used. An actively heated iridium afterheater and a lid with an opening were placed on top of the crucible. Crystal growth occurred at a rate of 0.5 mm h^−1^ and a rotation rate of 10 rpm.

**Figure 1:**
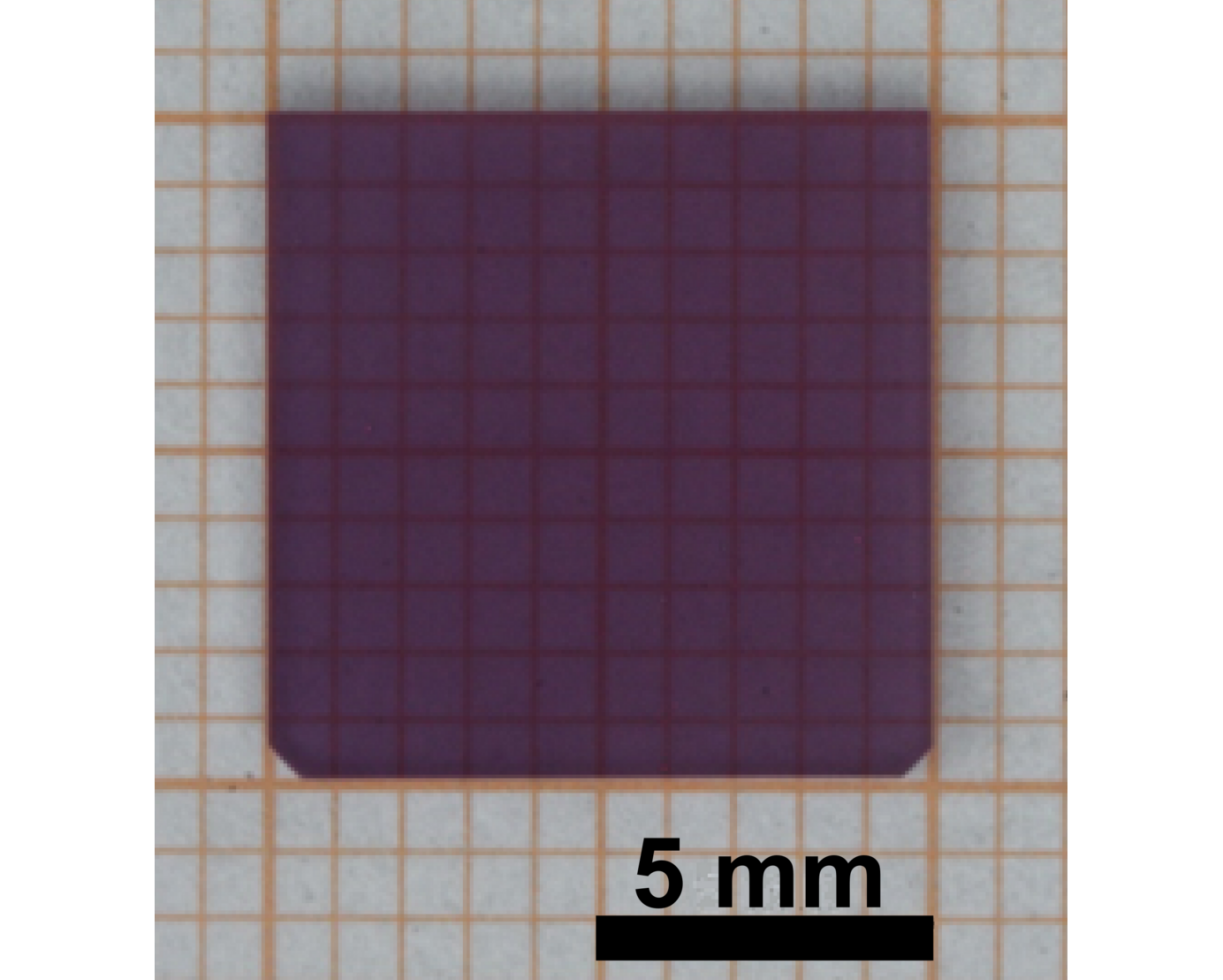
Typical one-side polished NSAT sample prepared from a Czochralski grown crystal with a starting composition of Nd_0.396_Sr_0.604_Al_0.698_Ta_0.302_O_3_. The composition of the grown crystal is expected to contain a few mol% more Nd than the melt from which it was grown.

The grown NSAT crystals were suitable for the preparation of large samples for measurements in various geometries to investigate their material properties by the characterization techniques described in the following paragraphs. Fig. 1 ▸ shows an example of a one-sided polished sample with a surface area of about 100 mm^2^. Due to compositional segregation during growth, it is likely the samples used are a few mol% more Nd rich than the initial composition, but this is a very approximate estimate. Nonetheless, this is not considered to significantly affect the measured properties and the conclusions obtained here are valid for a wider range of NSAT compositions since the properties, such as overall structure, vibrational modes and, especially, detail in which they are described do not depend so strongly on the exact composition of the material. Fine details, such as peak intensities or exact lattice parameter will change with composition, however, this is not the focus of this study.

### 2.2. XRD

#### 2.2.1. Powder X-ray diffraction measurements (PXRD)

PXRD were performed with a Stoe Stadi MP diffractometer with an Mo X-ray source (λ = 0.71073 Å) fitted with a curved Ge (111) monochromator and a DECTRIS MYTHEN detector in a modified Debye–Scherrer geometry with the sample prepared as a thin coating between two acetate films. Diffraction patterns were measured over a range of 2–84° 2Θ (scattering vector range, *Q* = 0.3–11.8 Å^−1^) with sampling resolution of 0.005° 2Θ for a duration of 0.5 h per data point. The samples were prepared by milling small amounts for Czochralski grown NSAT crystal into a powder using an agate mortar.

Rietveld refinements were performed using *GSAS-II* software (Toby & Von Dreele, 2013 ▸). The starting crystal structure was a generic *AB*O_3_*Pm*3*m* cubic perovskite that was then doubled to a 2 × 2 × 2 supercell and atomic identities ascribed to Nd_0.4_Sr_0.6_, and Al_0.7_Ta_0.3_ for the *A* (0, 0, 0) and *B* (½, ½, ½) sites of the perovskite subcell. The mean squared thermal displacement was assumed to be isotropic and was initially set to 0.01 Å^2^. Next, the *Fm*3*m* perovskite structure was refined for unit cell, atomic occupancies of the two Al/Ta sites and thermal displacements of all atoms. Constraints were applied to ensure the structure matches to the assumed chemical composition (growth composition rounded to the first significant digit) of the sample (Nd_0.4_Sr_0.6_Al_0.7_Ta_0.3_O_3_). The background was approximated using Chebyshev polynomials, and the instrument function was empirically fitted by refining the Caglioti function parameters with included asymmetry. Sample absorbance was set to 1 based on theoretical estimations (Von Dreele *et al.*, 2013 ▸). Unit-cell modifications and visualization was assisted by *VESTA 3* software (Momma & Izumi, 2011 ▸).

#### 2.2.2. Single-crystal X-ray diffraction (SC-XRD)

SC-XRD was measured using a Bruker D8 Venture diffractometer. It employs a KAPPA four circle-goniometer equipped with an X-ray microfocus tube with an Mo anode (λ = 0.71073 Å) and a Photon II 7 charge-integrating pixel array detector, as well as a multilayer monochromator. Larger NSAT-pieces from, both the shoulder area and the boule itself, were crushed using an agate mortar and suitable fragments were selected for subsequent study under a microscope. For the measurement, these were then mounted on a Microloop sample holder (Jena Bioscience, Ø = 100 µm) and fixed to the goniometer head. All experiments were conducted at room temperature and full diffraction spheres up to θ = 56.5° with maximum resolution of 0.65 Å (*Q* = 9.7 Å^−1^) were collected.

The frames were integrated using the *SAINT* software package (Bruker AXS Inc., 2021 ▸) using a wide-frame algorithm. To correct for absorption effects, the multi-scan method (*SADABS*) was used. By evaluating the systematic extinctions using *XPREP* (Sheldrick, 2008 ▸), the space group *Pm*3*m* could be determined. For further structure solution and refinement, the *SHELX* suite (Sheldrick, 2008 ▸) implemented into *OLEX2* was used. The displacement parameters were refined isotropically and towards the end of the refinement, a weighting scheme was applied.

### 2.3. Transmission electron microscopy (TEM)

Two TEM samples of the NSAT crystal were prepared with the surfaces normal to the [100] and [110] directions (the directions are the same for both, *Pm*3*m* and *Fm*3*m*, unit-cell descriptions) by polishing on diamond lapping films and final argon ion milling to electron transparency with 5 keV and an incident angle of 4° followed by stepwise reduction of the ion energy down to 200 eV and increased incident angles up to the final angle of 8°.

Scanning transmission electron microscopy (STEM) images were recorded with an FEI Titan 80-300 TEM equipped with an imaging C_s_-corrector and a high-angle annular dark field (HAADF) detector. The semi-convergence angle was set to 9.3 mrad and a camera distance of 196 mm was used. Due to charging of the sample the dwell time was set to 0.5 µs to avoid image distortions. To enhance the signal-to-noise ratio (SNR) a series of 16 images were taken and averaged after cross-correlation to eliminate image shifts.

Further HAADF-STEM data were collected from a lamella prepared by focused ion beam. The microscope used was a probe-corrected Thermo Fisher Spectra 300, operated at 300 kV, with a semi-convergence angle of 25 mrad, a camera length of 115 mm and a current of ∼100 pA. Energy-dispersive X-ray spectroscopy (EDXS) maps were acquired using a Dual-X detector setup with a total collection solid angle of 1.7 sr, with a current of ∼300 pA.

For STEM image simulations a parallelized multi-slice code based on the Kirkland code was used (Kirkland, 2020 ▸). The parameters used for the simulations were: acceleration voltage 300 kV, spherical aberration C_s_ 1.2 mm, semi-convergence angle 9 mrad, defocus 48.6 nm, sampling 2048 × 2048 pixel, slice thickness 0.54344 nm, scan sampling 26 × 22 probe positions in an area of 1.38 nm × 1.15 nm, and ten frozen phonon approximations for a temperature of 300 K and Debye–Waller factors of 0.076. For the positions with mixed atom types one actual type was randomly chosen, weighted by the occupation values for the atom types of that position. The simulated HAADF signals were integrated from 40 mrad to 280 mrad and the 26 × 22 scan point images where then scaled up to have the same pixel size as in the experiment.

### 2.4. Ellipsometry

Infrared (IR) spectroscopic ellipsometry was performed using a Fourier-transform ellipsometer (Woollam IR-VASE) over a range from 250 cm^−1^ to 6000 cm^−1^ (≃ 0.75 eV) whereby the spectral resolution was set to 4 cm^−1^. Visible and ultraviolet (UV–vis) spectroscopic ellipsometry was performed using a scanning variable-angle spectroscopic ellipsometer based on a grating monochromator operational in the spectral range between 0.5 and 6.5 eV with a step-size of 10 meV. The instrument is equipped with an autoretarder. Both measurements in the IR and UV–vis spectral region were taken at three different angles of incidence Φ = 50°, 60° and 70°. Additionally, the transmission spectrum was determined using the same UV ellipsometer device in the same spectral range and resolution (for transmission Φ = 0° with sample between the source and the detector). All measurements were performed at room temperature.

Ellipsometry yields the amplitude ratio between the parallel and perpendicular component of the reflected polarized light from the sample with respect to the plane of incidence (Ψ) and the corresponding phase shift between these components Δ. Ψ and Δ can be converted directly into the pseudo dielectric function 〈ε〉. As the sample in this study was a bulk material, 〈ε〉 in the IR region is nearly identical to the actual dielectric function (DF) ɛ. The IR-DF yields the transversal optical (TO) IR-active phonon modes. Due to the cubic crystal structure of NSAT, the DF is isotropic. To consider minor differences caused by the different angles of incidence, a model-based evaluation was nevertheless performed. For this, a model based on the well established multi-phonon sum (Cuscó *et al.*, 2022 ▸; Kluth *et al.*, 2023 ▸; Feneberg *et al.*, 2016 ▸), shown in equation (1) was used. This was a starting point for a point-by-point (pbp) fit, a numerical fit to the experimental data at every wavenumber until the best match was achieved. Afterwards, the model was applied again in equation (1) to line-shape fit the pbp-DF to gain the wavenumber values of the IR-active phonon modes.
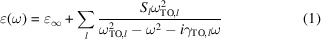
The dielectric loss function can then be calculated from the DF as shown in equation (2):
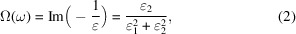
where ɛ_1_ and ɛ_2_ are the real and imaginary parts of the DF, respectively. In the UV–vis region the approach is similar, however, the surface roughness has to be considered as well. A multi-layer model is used with an effective medium approximated layer (EMA) using Bruggeman’s formalism (Bruggeman, 1937 ▸) (≃13 nm) on top of the bulk layer where a parametric General oscillator model (WVASE 32; Woollam, 2008 ▸; Craig *et al.*, 1998 ▸) is used to fit the experimental data followed again by the pbp-fit.

### 2.5. Vibrational spectroscopy

#### 2.5.1. Raman spectroscopy

All Raman investigations were conducted using (100)-oriented NSAT wafer samples at room temperature (298 K). To ensure the appropriate surface quality, the investigated wafers were polished using a motorized polishing disc with 1 µm as the final grit size. For all Raman measurements, a Horiba LabRAM HR Raman microscope equipped with a green laser (λ = 514 nm, 10 mW) and Zeiss Jena objectives (10×, 50× and 100× magnification) was used. The laser beam was focused onto the sample under 50× and 100× magnification through vertical alignment. All measurements were performed using a 100× objective with an aperture diameter of 250 µm and 100% laser power. Comparing measurements with lower laser powers did not show a noticeable decrease in the background intensity in comparison to the peak intensity. The background was fitted and subtracted from the measured spectra using an algorithm as presented by Eilers & Boelens (2005 ▸). The peak fitting procedure implemented the *lmfit* package (Newville *et al.*, 2025 ▸) based on a pseudo-Voigt function to accurately capture the Raman peak profiles.

#### 2.5.2. Infrared spectroscopy

IR spectroscopy was performed with a powdered NSAT sample made by crushing and grinding a small part of NSAT single crystal in an agate mortar. IR spectra of the powdered samples were measured using Thermo Scientific iS5 Fourier transform infrared (FT–IR) spectrometer with a resolution of 4 cm^−1^ at room temperature. Measurements were performed in transmission, in the form of a KBr pellet using an iD1 transmission accessory, as well as in attenuated total reflectance (ATR) mode using an iD5 single reflection ATR accessory with a Ge prism. 128 scans were averaged for transmission and 288 for ATR measurements. Such a high number of scans was needed as the signal was greatly reduced due to IR light scattering from air bubbles and NSAT particles, which are presumed to have noticeably higher refractive index than KBr. The Ge-ATR prism has a very low interaction volume due to the high refractive index of Ge, hence the SNR of a single scan is very poor. The transmission IR spectrum showed very significant background that was correct by subtracting a very large and broad Gaussian peak centred around 4000 cm^−1^ with width of near 10000 cm^−1^. ATR–IR spectrum data fitting was performed by multiple Voigt peak least-squares minimization using Levenberg–Marquardt algorithm (Press *et al.*, 1992 ▸) implemented in *Igor Pro 9* package (Wavemetrics Inc., 2023 ▸).

## 3. Results

### 3.1. XRD

**Figure 2:**
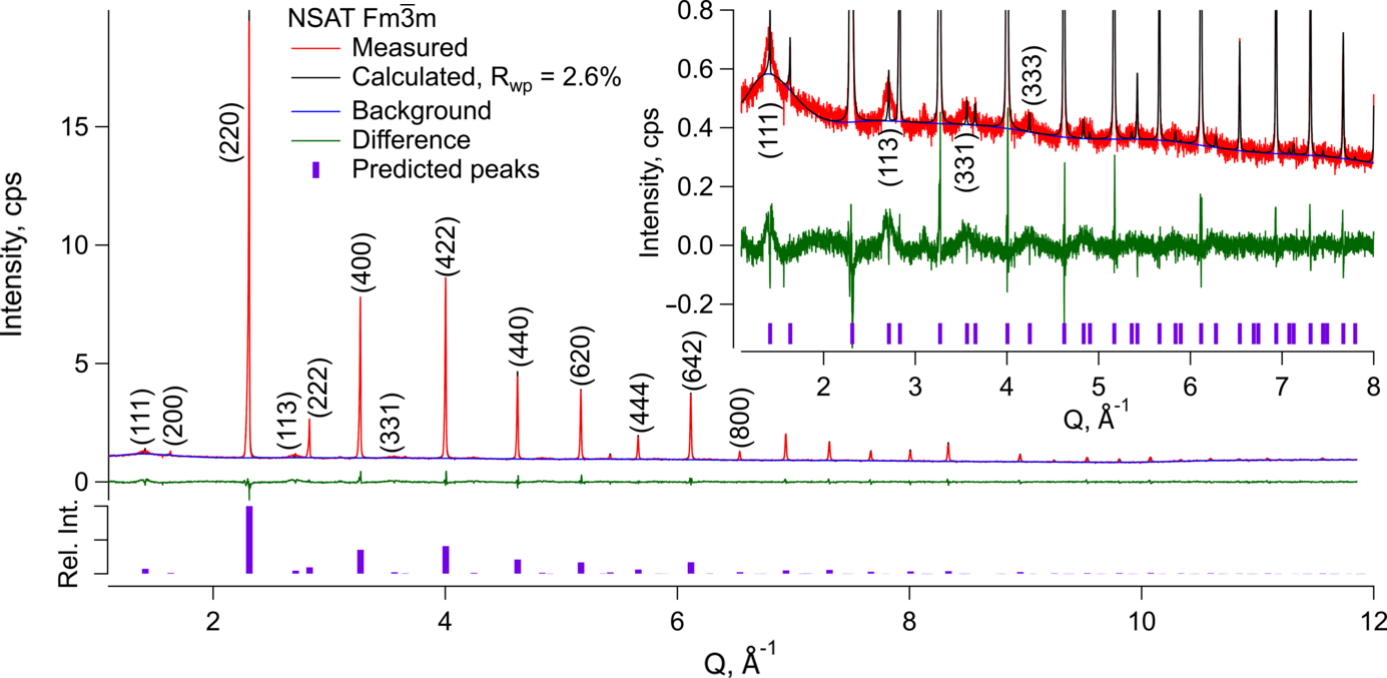
Powder XRD pattern in counts-per-second (cps) versus scattering vector *Q* of a Czochralski grown NSAT sample of Sr_0.6_Nd_0.4_Al_0.7_Ta_0.3_O_3_ composition together with a refinement using an *Fm*3*m* symmetry unit cell. Indexing of selected peaks and their relative predicted intensities are also shown.

Powder XRD of the NSAT sample showed a series of sharp peaks, which, although mostly consistent with a basic cubic perovskite of *Pm*3*m* symmetry, are best described by the *Fm*3*m* structure as it accounts for all, not just the most apparent peaks (see Fig. 2 ▸). *Pm*3*m* symmetry cell can only explain the sharp, high intensity reflections. A series of broad peaks was observed, consistent with locations where some or all *hkl* indices of the *Pm*3*m* structure would have a half-integer value. Considering the very different peak profiles of the two sets of reflections it was assumed this arises due to a superstructure ordering of some type on one of the two cation sites. To explore this, the standard cubic perovskite *Pm*3*m* unit cell was doubled in all three directions and all symmetry was removed creating a pseudo-cubic *P*1 triclinic cell. Nd/Sr and Al/Ta occupancies were refined under a constraint that the total chemical composition is conserved. The result was no apparent ordering on the Sr/Nd sites and a very clear formation of Ta rich planes normal to the (111) direction. As this was the same type of superstructure ordering to that observed in *Fm*3*m* LSAT (Li *et al.*, 2003 ▸), the symmetry of the unit cell was determined to be *Fm*3*m* with a doubled lattice parameter relative to the basic *Pm*3*m* perovskite (see Fig. 2 ▸).

Refinement using *Fm*3*m* symmetry generated a very close match to the observed pattern (*R*_wp_ = 2.6%). The lattice parameter was found to be *a* = 7.6856 Å. No systematic deviations were found in the residual after refinement, aside from the poor fit of the peak profile of the superstructure peaks. This results because of the very different domain size of the overall material (bulk single crystal) and the superstructure domains (nanoparticulate). Scherrer’s formula, when applied to the (111) peak, yields a domain size of about 5.5 nm. This is consistent to the observations in TEM described below. The site occupancies of the two Al/Ta sites is refined to Al/Ta = 0.6/0.4 versus 0.8/0.2 fractional occupancy. Note that the degree of Ta segregation to one site is almost certainly underestimated as the superstructure peak area is not well fitted due to its broad profile (see the inset in Fig. 2 ▸).

Processing the data obtained through SC-XRD experiments initially yielded a cubic unit cell with *Pm*3*m* symmetry and lattice parameter of *a* = 3.841 (4) Å, thus confirming the material to be a cubic perovskite. Nevertheless, a great number of samples (>10) was needed to be measured to obtain a suitable dataset. It became apparent, that samples obtained from the shoulder piece of the single-crystal boule were less marred by strain and other defects thus resulting in measurement data of higher quality; some quality issues were always present, however.

**Figure 3:**
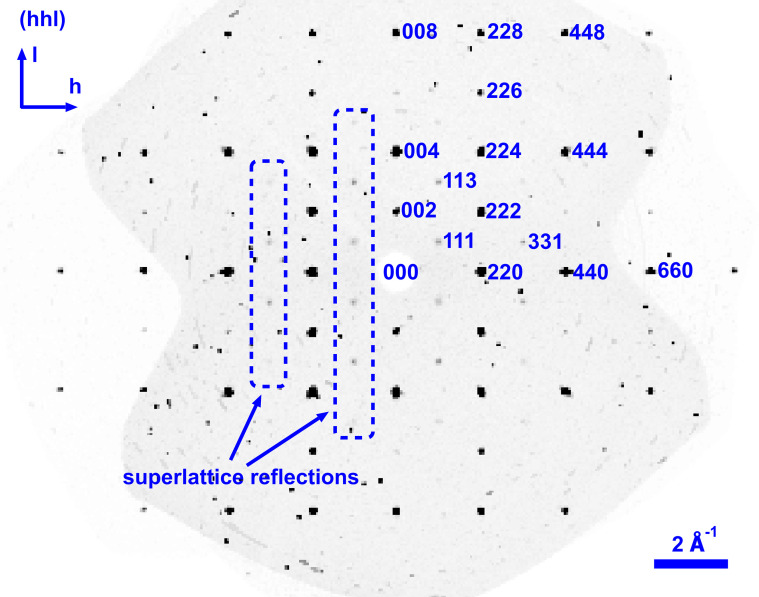
Precession image (*hhl*) zone, created from a total of 4182 detector frames showing superstructure reflections (blue boxes) at odd-integer *hkl* values corresponding to the *Fm*3*m* perovskite unit cell (or half-integer *hkl* if the *Pm*3*m* description is used). As the *Fm*3*m* symmetry applies only due to superstructure ordering, the underlying basic structure being *Pm*3*m*, the main lattice reflections have only even *hkl* indices, while superstructure peaks have odd *hkl* indices.

The diffraction image showed additional reflections that were not explained by the *Pm*3*m* symmetry (see Fig. 3 ▸), which therefore originate from the superstructure. Besides the superstructure reflections, the precession images along different directions exemplify the presence of a plethora of other parasitic reflections in the material, which were observed in all samples to varying amounts, presumably due to small crystalline particles chipping off the surface and powder-like ring fragments, likely of similar origin. Again, the samples from the shoulder piece proved to be of better quality.

An attempt to solve and refine the structure in the *Pm*3*m* space group using *SHELX* was made by populating the *A*-site with Nd and Sr and the *B* site with Al and Ta. During fitting, the superstructure reflections were omitted. Table 1 ▸ presents the thus obtained results upon convergence. Fitting the atomic occupation of the *B*-site proved to be difficult, as exemplified by the drastic overestimation of the Ta-concentration. Beyond that, the comparatively high weighted residuals (*wR*_2_) implies that the model does not fully account for the majority of (in particular weaker) reflections, leaving a high residual electron density.

Employing a face-centred space group *Fm*3*m* (see Table 1 ▸) results in a higher fit quality, thus inferring an improved coverage of the measured reflections. Nevertheless, the refined occupancies overestimate the Ta-concentration on both the *A* and *B*-sites, leading to the total cation charge of the calculated structure being about 10% higher than nominal. This overcompensation and the comparatively minor improvement of goodness-of-fit (GoF) indicates, that the description by *F*- or *P*-centred unit cells alone is insufficient to comprehensively resolve and account for all features that the superstructure introduces into the electron density of the crystal. Furthermore, localized accumulations of residual electron density appear systematically in the vicinity of the oxygen and the *B*-cation site, indicating a degree of disorder on these particular sites.

### 3.2. TEM

**Figure 4:**
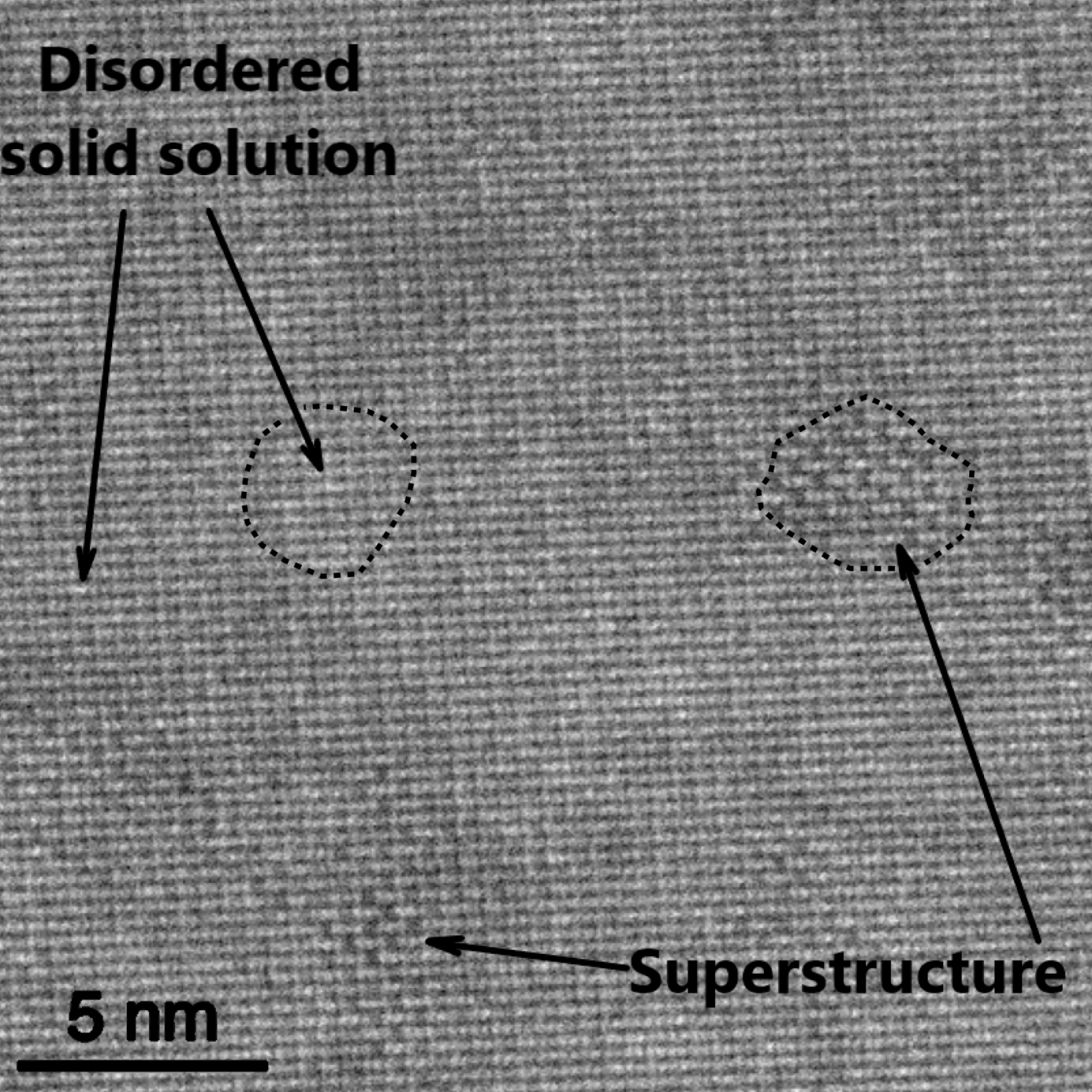
STEM image of NSAT in [110] projection. In between the regular alloyed structure, a pattern of pronounced superstructure contrast on {111} planes is observed, which extends for a few nanometres. Representative areas of the superstructure and the apparent solid solution are highlighted by dashed lines.

In the [100] projection the STEM images displayed no superstructure ordering consistent with a solid solution nature of NSAT. In contrast, image patterns of the [110] projection exhibited irregular shaped areas with a superstructure pattern on {111} planes at the Al/Ta column positions and of varying contrast strength (Fig. 4 ▸). Although the observed superstructure pattern is in accordance to the superstructure found by X-ray refinement, the contrast in the STEM patterns of the superstructure is stronger than one would expect from the mean atomic number (*Z*) value per unit length in the [110] projection.

To explain this discrepancy, STEM image patterns of three different structure models were simulated:

(1) the disordered (*Pm*3*m*) case of Nd_0.4_Sr_0.6_Al_0.7_Ta_0.3_,

(2) the superstructure obtained from the PXRD refinement, and

(3) a superstructure with increased Ta segregation, where the Al/Ta columns on {111} planes are either pure Al or Al_0.4_Ta_0.6_, the maximum possible segregation of Ta within the superstructure of average composition of Al_0.7_Ta_0.6_. To better compare with the experimental images, an SNR of 4 was applied.

**Figure 5:**
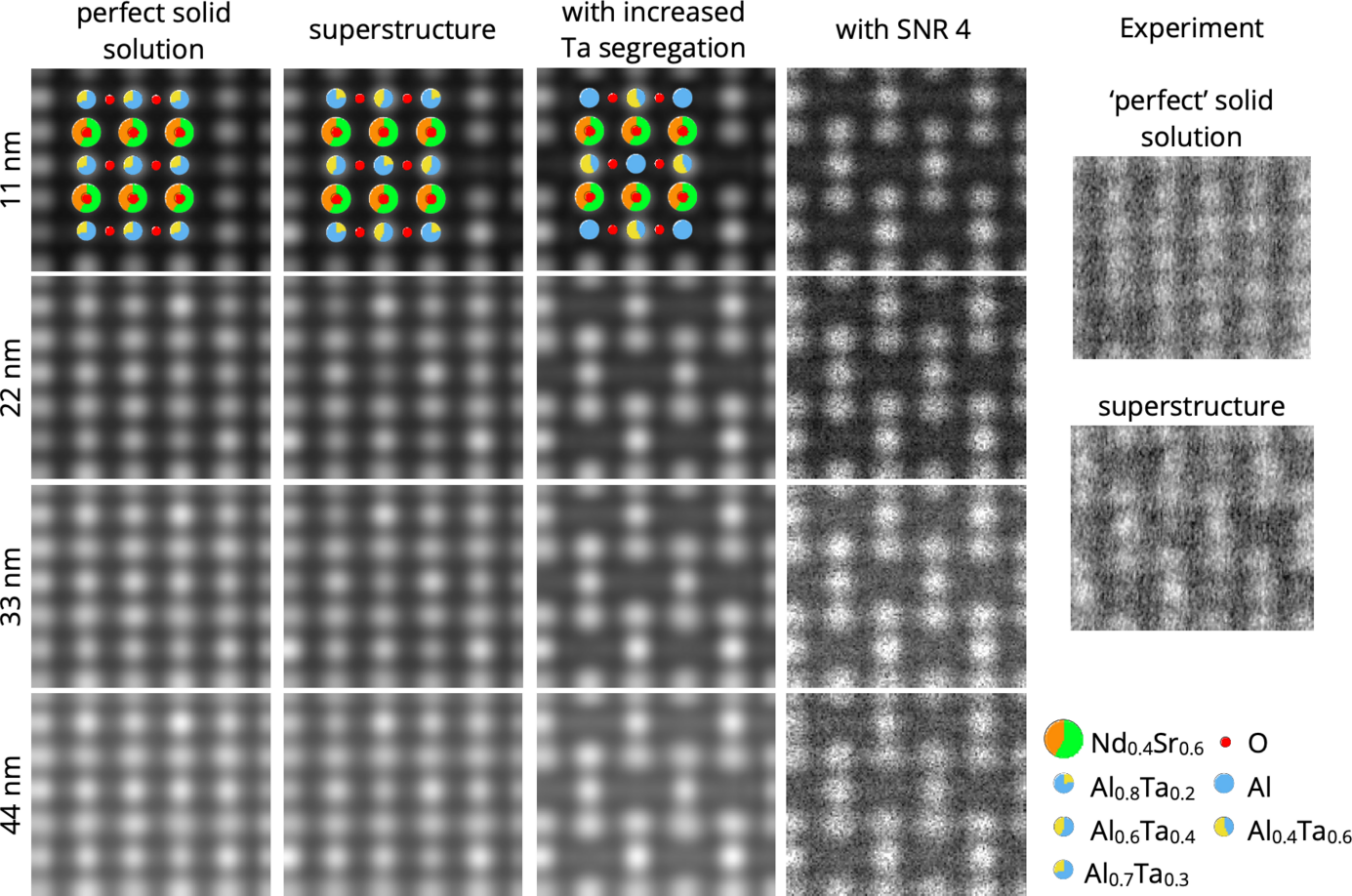
STEM image simulations showing a comparison between NSAT as an ideal solid solution, the refined superstructure from PXRD, a superstructure developed to its maximum extent, the same superstructure with SNR of 4 applied and observed experimental patterns. The overlays show the simulated structure models.

For all simulations a supercell of 4 × 6 × 50 along [110] × [001] × [110] was constructed. The simulation results at different thicknesses are shown in Fig. 5 ▸ beside the experimental patterns with and without superstructure contrast.

When comparing the simulations with the observed superstructure pattern, the contrast of the superstructure with increased Ta segregation resembles the contrast in the experimental images where the superlattice contrast is strongest. Furthermore, the contrast pattern of the more segregated superstructure has a similar appearance over a range of thicknesses as observed in STEM experiment, while the pattern of the less segregated superstructure is similar to the pattern of the perfect solid solution for higher thicknesses. Therefore, the degree of superstructure ordering observed in STEM is locally higher than that calculated form PXRD and is close to the maximum Ta segregation that is achievable in NSAT of this bulk composition.

**Figure 6:**
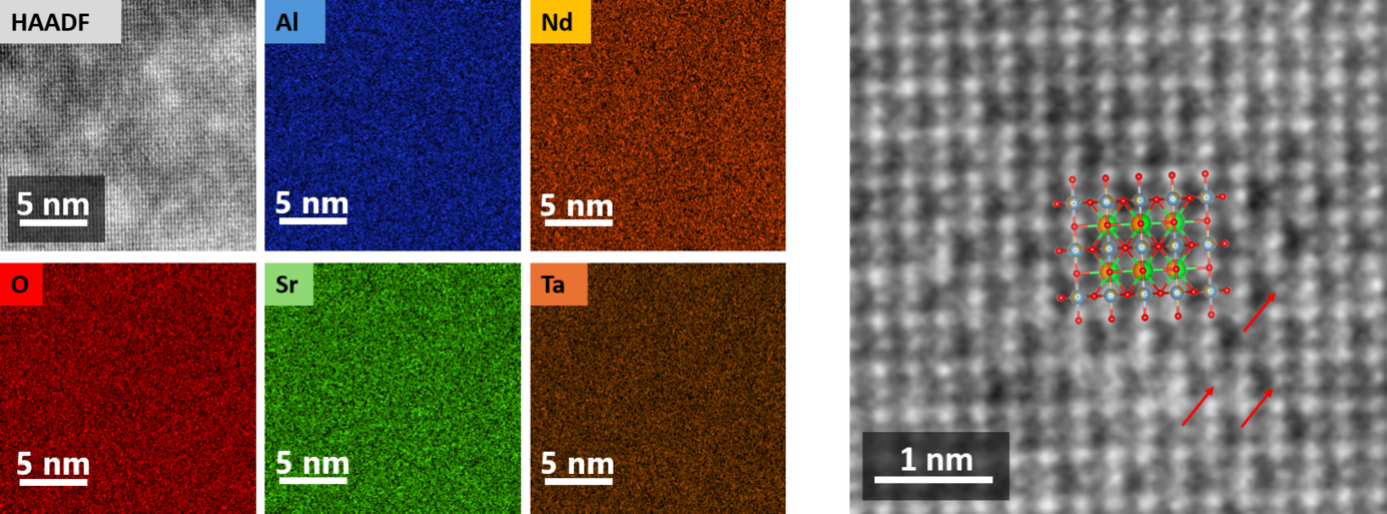
STEM-EDXS compositional maps (left) acquired in the [110] direction in a region presenting superstructure contrast, showing a homogeneous elemental distribution. HR-HAADF-STEM image (right) showing the local superstructure contrast, with the corresponding arrangement of the model. Red arrows highlight some off-centre atomic columns. In the model Nd is orange, Sr is green, Al is blue, Ta is brown and O is red, same as in the EDXS maps.

Additional examination using a STEM equipped with EDXS reveals the chemical composition is uniform in the NSAT sample and no segregation is found (Fig. 6 ▸). HAADF-STEM imaging performed together with STEM-EDXS shows that some atomic columns are displaced from their ideal symmetrical position (Fig. 6 ▸ right, indicated by red arrows). As this effect is not clearly visible in the X-ray analysis, it is probably not a recurrent structural feature. It could be caused by physical effects in the original sample or be an artefact due to sample preparation. As it does not affect crystallography at medium range and, therefore, the effectiveness of NSAT as a substrate, it will be investigated at a later time.

### 3.3. Ellipsometry and vibrational spectroscopy

#### 3.3.1. Ellipsometry

**Figure 7:**
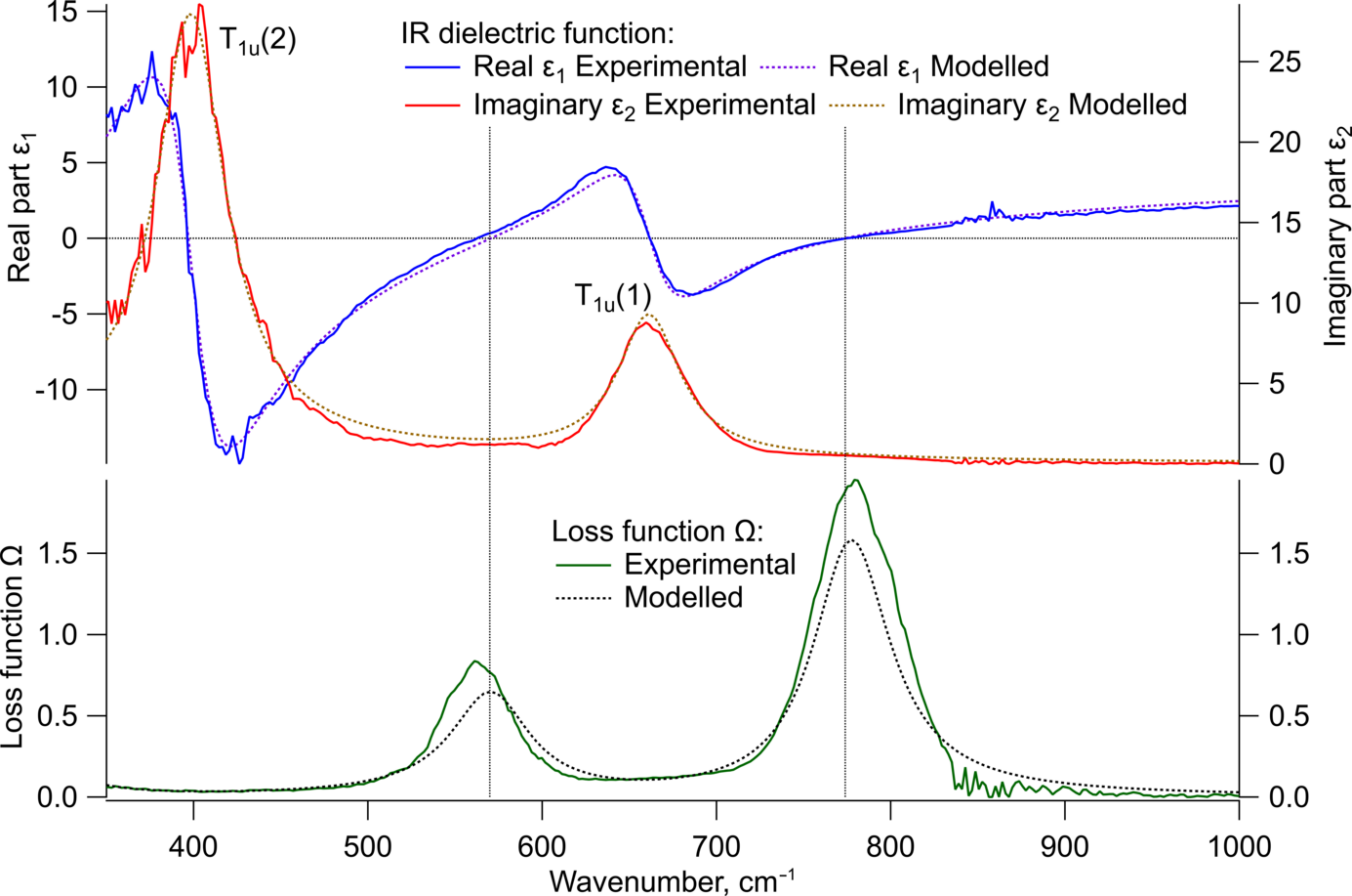
Infrared dielectric function with its real part (ɛ_1_) in blue, the imaginary part (ɛ_2_) in red, and the loss function based on equation (2) in green compared to the corresponding models in dotted lines. A horizontal line at zero in and two vertical (dotted) lines are added to show the positions of the second zeros in the real part which correspond to the maximum in the loss function.

Two IR-absorption modes are identified in the IR-ellipsometry experiment near 660 cm^−1^ and 400 cm^−1^ (see Fig. 7 ▸). The observed modes were named T_1u_(1) and T_1u_(2), starting at high wavenumbers. The peaks in the loss function match the second zeros in the real part (marked by vertical dotted lines), where the corresponding longitudinal optical (LO) phonon modes would be expected. The model fitting of the experimental data refined the two IR absorption bands to 662 cm^−1^ and 399 cm^−1^ (see Table 2 ▸). The match between model and experimental loss function also confirms the *ɛ_∞_* of 4.0.

**Figure 8:**
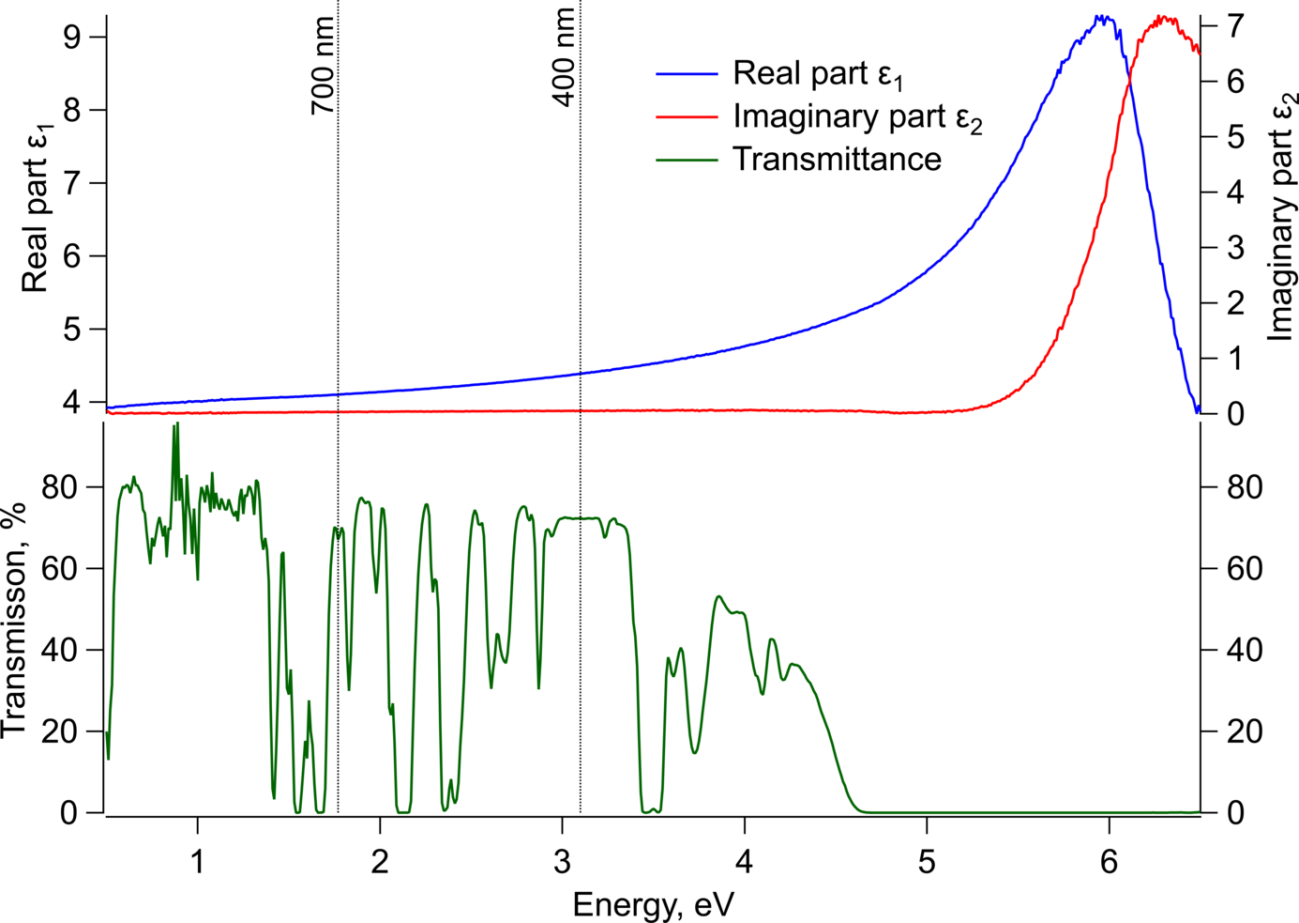
UV–vis range dielectric function with its real (ɛ_1_, blue) and imaginary (ɛ_2_, red) parts and the measured UV–vis transmittance in green. 700 and 400 nm dotted lines are added as a guide to indicate the visible part of the spectrum.

The experimental UV-DF with its real part corresponding to the square of the refractive index, and the imaginary part to the absorption coefficient is displayed in Fig. 8 ▸. The initiation of the strong absorption is at about 5.2 eV. Additionally, the transmission *T* is shown in comparison to ɛ_2_. *T* undergoes notable changes, fluctuating between 0 and 0.8 before eventually dropping to zero at around 4.6 eV. This behaviour is in agreement to with previous results by Pawlak *et al.* (2005 ▸). These sharp peaks in the transmission spectra suggest point-defect related absorption within the crystal. Pawlak *et al.* suspected the Peaks to be Nd^3+^ related since they are also observed in similar crystals containing Nd (Pawlak *et al.*, 2005 ▸).

The clear difference between a non-zero transmission and the absorption initiation of at least 0.6 eV leads to the assumption that the fundamental bandgap is to be found at ≃ 4.6 eV and is either indirect or direct but dipole forbidden. The absorption initiation in ɛ_2_ could thus be associated with the first direct dipole-allowed transition.

#### 3.3.2. Vibrational spectroscopy

**Figure 9:**
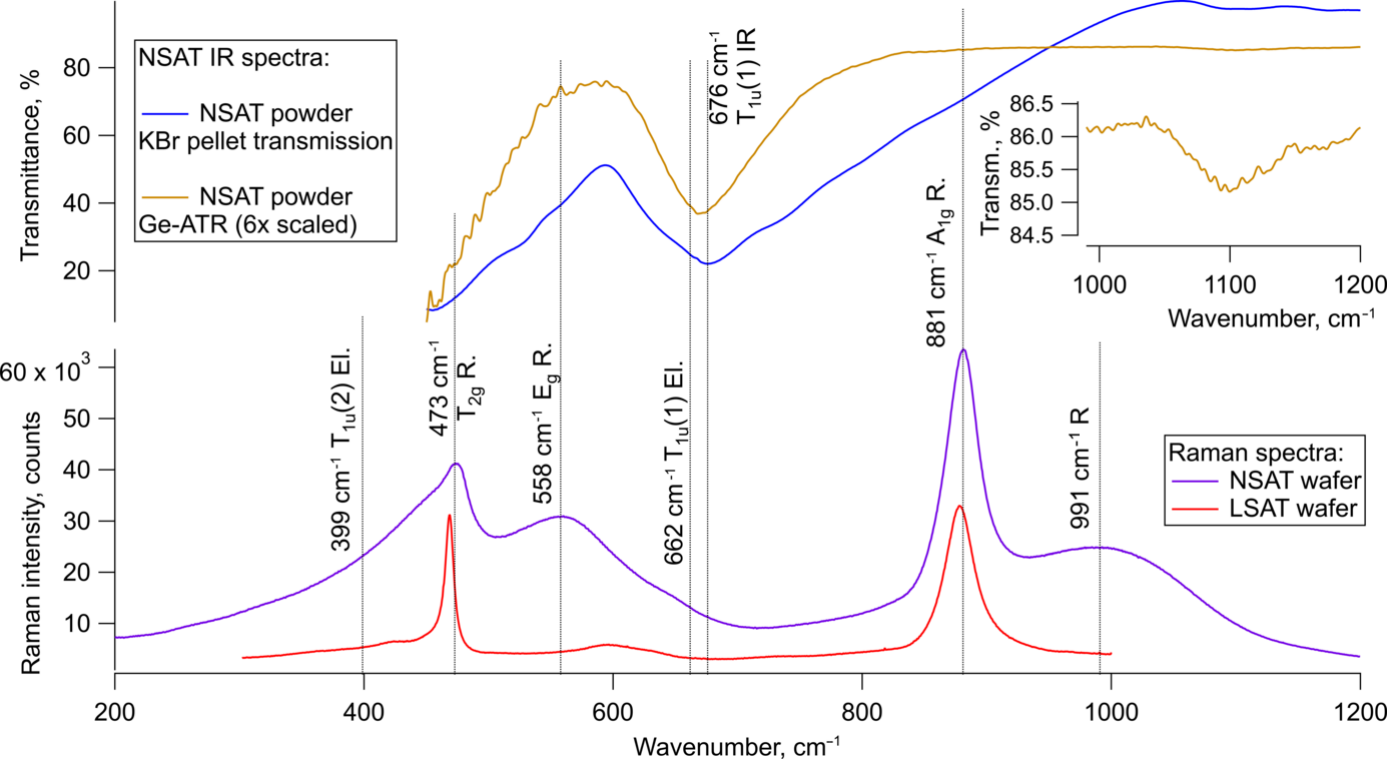
Raman spectroscopy on NSAT wafer and IR spectroscopy results on NSAT powder samples. In band assignments El, R and IR represent ellipsometry, Raman and IR spectroscopy as the method used to identify the vibrational mode. An unexpected mode at 991 cm^−1^ can be observed. A Raman spectrum of an LSAT wafer is also added for comparison. Due to the weak signal of Ge-ATR its signal has been enhanced six times. A Gaussian background has been subtracted from the transmission IR spectrum due to enormous scattering effects believed to be Mie scattering of micron scaled NSAT particles and air bubbles dispersed in KBr. The inset shows a magnified weak peak to highlight its presence in the Ge-ATR measurement.

**Figure 10:**
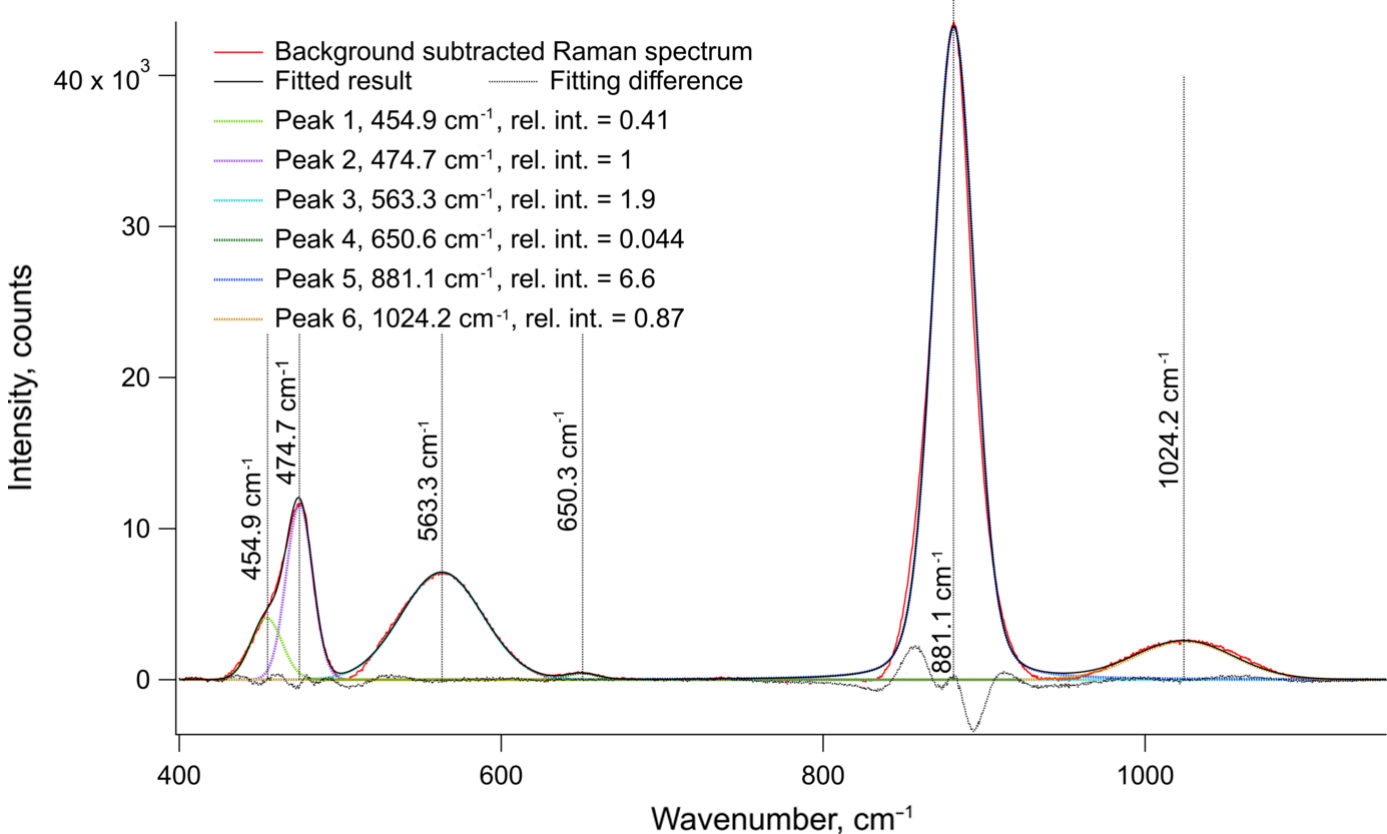
Peaks extracted from the deconvoluted and fitted Raman spectrum of a (100)-oriented NSAT-sample. Peaks of uncertain origin, such as 1024.23 cm^−1^ and 454.91 cm^−1^ can be seen as can the forbidden T_1u_(1) peak at 650.61 cm^−1^. Due to peak breadth and overlaps, the frequencies of fitted Voigt peaks do not fully match those observed with IR.

Unpolarized Raman spectra of NSAT are shown in Fig. 9 ▸. A strong, fairly sharp peak was observed at 881 cm^−1^ and slightly less intense, rather broad peaks were observed at 991 cm^−1^, 558 cm^−1^, 473 cm^−1^ and 399 cm^−1^. The peak at 399 cm^−1^ was observed to be highly asymmetric. To better separate different contributions a deconvolution was performed (see Fig. 10 ▸). Here six peaks could be identified at 455 cm^−1^, 475 cm^−1^, 563 cm^−1^, 651 cm^−1^, 881 cm^−1^ and 1024 cm^−1^. It is likely the peak 455 cm^−1^ is an artefact appearing due to asymmetry of the peak at 474 cm^−1^. The small peak identified at 651 cm^−1^ can also be observed in IR spectra.

**Figure 11:**
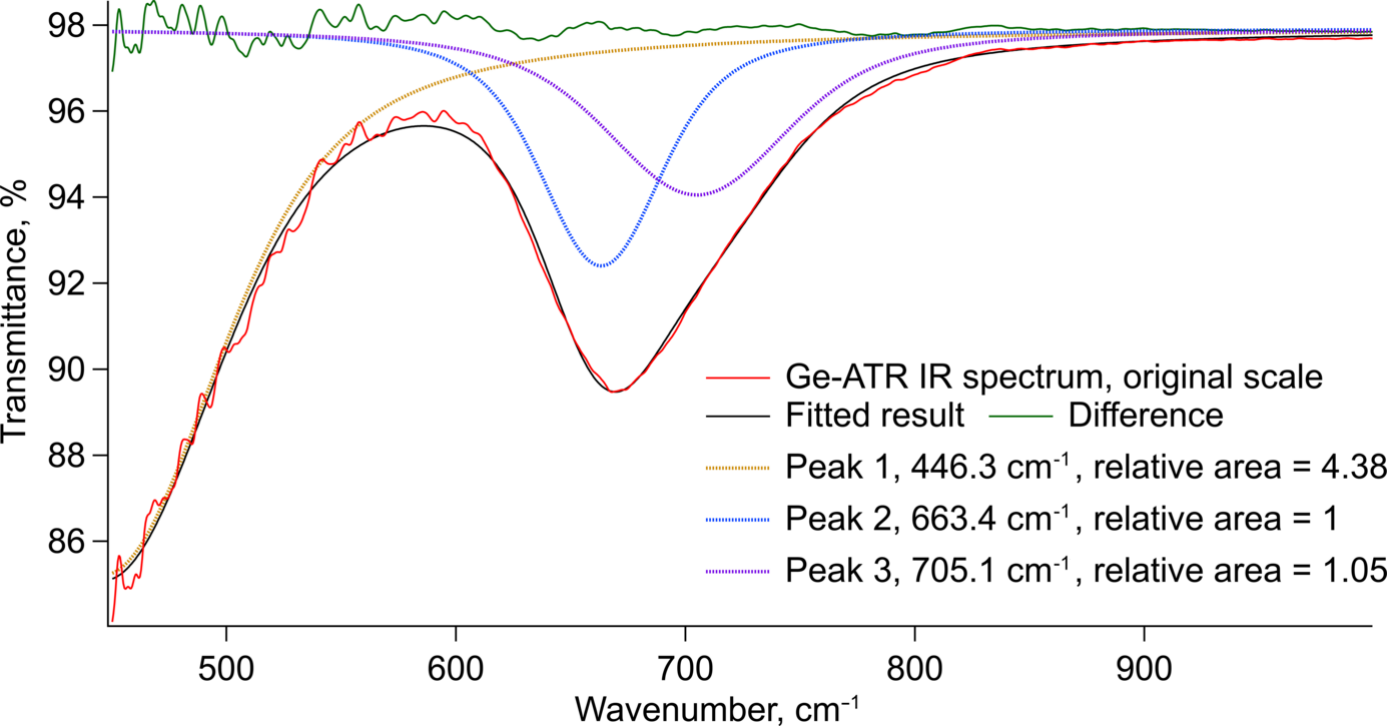
Ge-ATR IR spectrum fitting in the 450–1000 cm^−1^ range. Three peaks are identified, two of which are consistent with observations from ellipsometry, while an extra peak 705 cm^−1^ is also identified.

The IR spectra of the powder samples measured with Ge-ATR and the KBr pellet are consistent with each other. The Ge-ATR seems to provide a more accurate description of the peaks but worse SNR ratio. The transmission IR spectrum suffers from the poor quality of the KBr pellet. Both samples clearly show only one peak (due to limited measurement range) at 676 cm^−1^ and seemingly a beginning of another peak, likely centred towards 400 cm^−1^. A very weak peak is also seen in both IR spectra at 1100 cm^−1^, however, the source is unclear. These observations are consistent with findings from ellipsometry and the two bands at 676 cm^−1^ and 400 cm^−1^ can be assigned to two T_1u_ normal modes. Peak fitting of the Ge-ATR IR spectrum (see Fig. 11 ▸) suggests the T_1u_ band at 676 cm^−1^ could actually consist of two overlapping bands. This could, however, be due to overfitting of a single asymmetric peak. The presence of either asymmetry or a second peak indicate some underlying structural complexity is present (see *Discussion* 4.2).

## 4. Discussion

### 4.1. Crystal structure

PXRD, SC-XRD and HR-TEM data, agree that NSAT structure is based on a simple *Pm*3*m* oxide perovskite, however, when observed in detail the material is fairly complex. The crystal structure is best described by a supercell of 2 × 2 × 2 *Pm*3*m* cells symmetry with two distinct Al/Ta sites resulting in a *Fm*3*m* symmetry unit cell.

The TEM imaging reveals that in some areas the superstructure ordering has developed to the near-maximum extent of Al/Ta distribution (no Ta on one site and 60 at% on the other). Both XRD methods show a lower degree of Al/Ta segregation (around Al/Ta 0.6/0.4 versus 0.8/0.2) on between the two sites. This is due to the superstructure ordering consisting of domains that are very small in size. Scherrer’s formula indicates the average domain size as 5.5 nm, largely consistent with HAADF TEM images showing similar or even smaller domains. This results in two very different sets of peak profiles for the main perovskite lattice peaks (*hkl* even) and superlattice peaks (*hkl* odd). Since two different peak profiles cannot be simultaneously fitted, the fit underestimates the total intensity of the superstructure peaks and hence the amount of Al/Ta segregation is also underestimated. Furthermore, due to domains being very small (5.5 nm is equivalent to about seven unit cells) with associated anti-phase domain walls being highly abundant. If a unit cell at the domain boundary is considered ‘disordered’ and the Scherrer’s formula is assumed to give the correct domain size of 5.5 nm, nearly a third of the whole sample volume is contained in domain walls. The scattering from or very near these boundaries will not add intensity to the superstructure ordering peaks thereby further reducing their intensity. PXRD was actually found to be more capable in analysing NSAT and provided better fit results than SC-XRD. This is considered to be due to the high amount of residual stress in the bulk crystal that readily induces fractures leading to even small NSAT pieces having a polycrystalline character. The stress affecting the SC-XRD measurements, is mostly likely due to the location very close to the bulk crystal surface where the SC-XRD samples were taken from; it is unlikely to significantly influence the results obtained with other methods presented here.

XRD provides a much more averaged view of the material and better describes the long-range order, while with the TEM highly short-range ordered local areas can be found. These regions, however, do not necessarily represent the material as a whole. The best single unit cell description is likely closer to what is found in TEM with segregation of Al/Ta being fairly close to maximal achievable of Al/Ta of 0.4/0.6 on one and 1/0 on the other site.

SC-XRD was found to struggle in obtaining a high-quality dataset of an NSAT crystal piece to sample features consistent with internal stress. PXRD refinement yielded peak broadening due to a microstrain near 1000 p.p.m. (empirical observations suggest perfect single crystals usually have a few 100 p.p.m., but for solid solutions up to a few thousand can occur) and did not show evidence of excessive internal stress. This would be consistent with high residual stress in a bulk crystal after growth that makes SC-XRD measurements difficult, but that can be relieved by grinding it into a powder.

A similar superstructure is also observed in very closely related LSAT (Li *et al.*, 2003 ▸) and (La,Sr)(Al,Nb)O_3_ (LSAN) (Ito *et al.*, 2002*a* ▸). In LSAT it has been suggested that superstructure ordered domains are dispersed within a superstructure disordered matrix (all within a perfectly ordered *Pm*3*m*) single crystal) as this is observed via direct TEM imaging and is consistent with dark-field TEM imaging (Li *et al.*, 2003 ▸). Here, a similar observation with HAADF-STEM imaging has been made (Fig. 4 ▸). The same type of superstructure ordering is also well known in Sr(Al_0.5_Ta_0.5_)O_3_ (SAT) (Chen *et al.*, 1995 ▸; Tao *et al.*, 1996 ▸), however, in this material the stoichiometry allows for sites with only Al and sites with only Ta being present. A direct interpretation of the STEM images might suggest that some volume of the sample consists of a few nm of large superstructure-ordered domains, while the rest is superstructure disordered. Nevertheless, it has to be considered that the superstructure ordering of the type present in NSAT can form two anti-phase domains (depending if (0,0,0) or (0.5, 0.5, 0.5) develops into the Ta rich site) and the thickness of the TEM lamella is unlikely to be thinner than 20–30 nm. Therefore, anti-phase domain overlap in transmission projection is very likely and can lead to cancellation of the observed superstructure signal. If the material were to consist of superstructure ordered domains in a disordered matrix, there would have to be a reason why some parts of the sample develop a superstructure ordering and some do not. The only such reason that is plausible here is chemical segregation, implying there is a miscibility gap in the phase diagram. We were unable, however, to discern any chemical segregation in STEM-EDXS mapping. Although atomic resolution EDXS was not achieved, the resolution is much higher than the domain size of 4–6 nm. This is consistent with observations in LSAT (Li *et al.*, 2003 ▸), although, the instrumentation and resolution used by Li *et al.* (2003 ▸) was sub-optimal and only a line scan, no mapping, could be performed.

The very high likelihood of overlapping anti-phase domains in combination with no observed chemical segregation strongly suggests that NSAT consists of small nanoscale domains in its entirety. The domains can form with one of two, initially symmetry equivalent, sites enriching in Ta, leading to two anti-phase domains that complicate interpretation of TEM images, unless the lamella studied by TEM could be made extremely thin.

**Figure 12:**
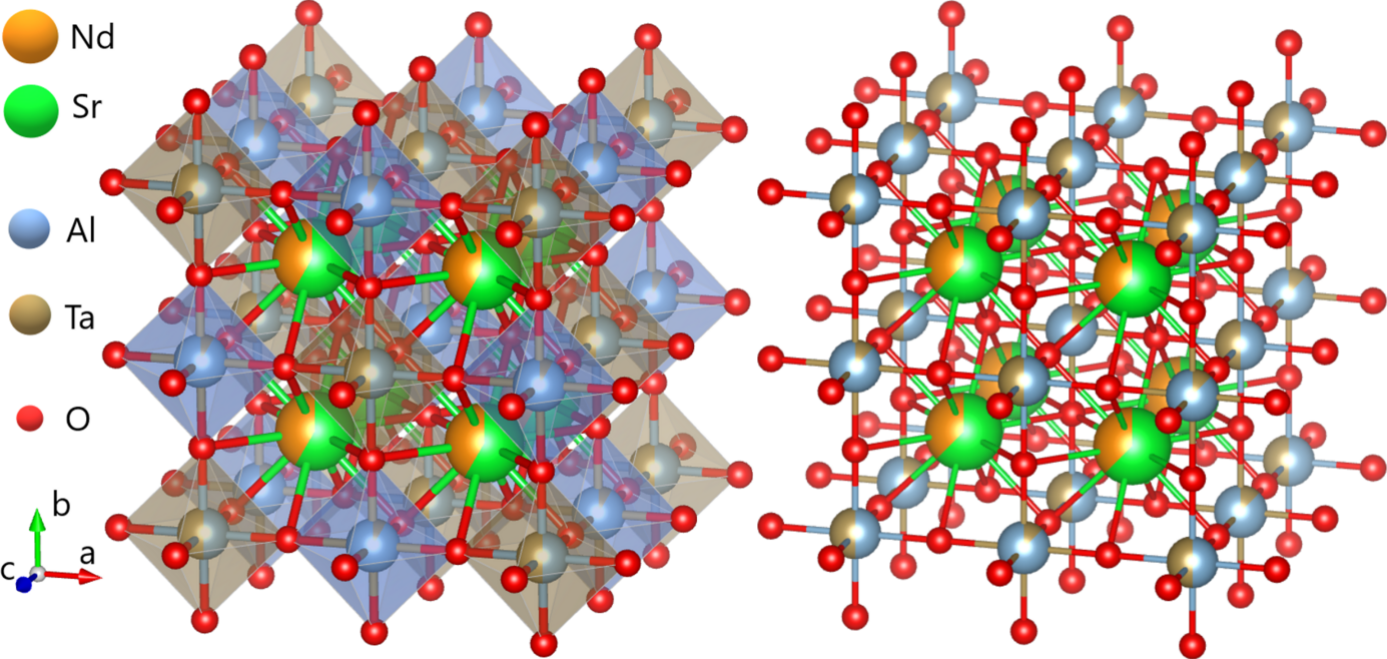
The *Fm*3*m* structure considered to be the most representative unit cell of NSAT with Al/Ta ratios of 0.5/0.5 and 0.9/0.1 on (0,0,0) and (½, ½, ½) positions, respectively, as visualized in *VESTA3* (Momma & Izumi, 2011 ▸). The (¼, ¼, ¼) positions are assumed to be equally occupied by Nd and Sr in 0.4/0.6 ratio.

The same superstructure ordering could also be explained by *I*4 symmetry (Steins *et al.*, 1997 ▸). No peak broadening, however, that could be consistent with a lower symmetry than cubic, such as *I*4 was observed. This implies that if NSAT is indeed a tetragonal symmetry material the distortion is so low it cannot be observed in a laboratory XRD experiment, even with a high-resolution Debye–Scherrer geometry instrument. Therefore, our conclusion is that as a bulk material NSAT is best described by a *Fm*3*m* symmetry structure and consists entirely of nanoscale ordered domains with short-range order developed close to maximal possible around Al/Ta 0.5/0.5 on one and 0.9/0.1 on the other site (see Fig. 12 ▸).

### 4.2. Lattice dynamics

The structure of NSAT is based on a cubic-perovskite *Pm*3*m* crystal structure with only minor changes induced by the superstructure ordering of Al/Ta into the overall bonding environment. Therefore, for the purposes of lattice dynamics analysis of NSAT here, it will be neglected. For space group *Pm*3*m* there are 12 optical phonon modes (Aroyo *et al.*, 2006*a* ▸; Aroyo *et al.*, 2006*b* ▸; Aroyo *et al.*, 2011 ▸; Kroumova *et al.*, 2003 ▸):
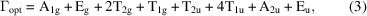
with the A_1g_, E_g_ and 2T_2g_ as the Raman active and only the 4T_1u_ as the IR-active modes. Two of the T_1u_ modes are observed in ellipsometry, one near 660 cm^−1^, the other around 400 cm^−1^. This correlates well with the IR spectroscopy where both modes are observed in transmission spectrum of the NSAT wafer. The higher energy peak at 660 cm^−1^ is also very clear in the two powder measurements, with ATR-IR suggesting it might even consist of two peaks. Some peak asymmetry also seems visible in the ellipsometry measurements. Since only a part of the 400 cm^−1^ peak can be seen in the powder IR measurements, due to limited measurement range of KBr optics equipped instrument, ellipsometry and IR cannot be directly compared. Due to the lack of literature data on this material it is not possible to compare the results with existing data from theoretical or experimental work. It is likely that only two (or potentially three) out of the four IR-active modes could be detected due to them lying below the lower wavenumber limit of the IR and ellipsometry instruments used in this study (450 cm^−1^ for IR spectroscopy and 250 cm^−1^ for ellipsometry). A very small peak identified in IR spectra at 1100 cm^−1^ is more likely to be an overtone or a combination band due to its low intensity, not an unaccounted T_1u_ mode. The 1100 cm^−1^ peak would match an overtone of the E_g_ mode as the frequency is very slightly less than double the E_g_ frequency of 563 cm^−1^, however, this would indicate a high degree of anharmonicity in the vibration and local symmetry deviations as overtones are IR forbidden in this space group.

Although no literature data on Raman measurements of NSAT are known, due to its structural and chemical similarity it can be compared to LSAT and SAT. The Raman spectrum observed for NSAT broadly matches both materials (see Table 3 ▸) with only slight shifts in band positions. A_1g_ (881 cm^−1^) and T_2g_ (474 cm^−1^) bands have slightly higher frequencies than in SAT or NSAT, while the E_g_ (556 cm^−1^) band has a lower frequency. Although plausible explanations on influences on different bands can be found in the literature (Tao *et al.*, 1996 ▸; Runka *et al.*, 2004 ▸; Ratheesh *et al.*, 2000 ▸), without detailed theoretical calculations the exact impact of cation substitution on different sites are somewhat speculative (NSAT adds even more complexity due to its partial superstructure ordering).

Both, SAT and LSAT (the comparison measurement performed in this study and literature (Runka *et al.*, 2004 ▸), show much sharper Raman peaks than NSAT. The peak intensity ratios are also different. The Raman spectrum of NSAT shows a very pronounced E_g_ band, which is very weak in the case of SAT and LSAT. Furthermore, there is an unassigned Raman peak at 991 cm^−1^, traces of which can also be observed in SAT and especially its Nb equivalent SAN (Tao *et al.*, 1996 ▸). The broad peaks together with altered intensities suggests a partially disordered material. This would be consistent with the XRD and TEM data that, although they show a near perfect *Pm*3*m* basic structure, also indicate the sample consists of nanoscale superstructure domains changing the overall symmetry to *Fm*3*m*. Due to the very small domain size each individual unit cell might have a lot lower symmetry when considered in isolation. This would also explain why a small peak around 650 cm^−1^ (T_1u_) could be detected in the Raman spectrum deconvolution analysis. In centrosymmetric materials IR and Raman activity are mutually exclusive and since this peak can be detected in both spectra it suggests some local reduction in symmetry and loss of the inversion centre. A similar peak in the Raman spectrum has also been observed for LSAT (Runka *et al.*, 2004 ▸). The double-peak nature of the T_1u_ band near 660 cm^−1^ indicated by the ATR-IR (see Figs. 9 ▸ and 11 ▸) also suggests some lowering of symmetry. Apart from the local cation disorder, stress from the growth process is also likely to be able to break local symmetry. Although not quantified here, it was qualitatively observed that issues consistent with residual stress are hampering SC-XRD measurements.

## 5. Conclusions

XRD and TEM show that NSAT has a very well defined oxygen network of a cubic perovskite with a lattice parameter of *a* = 3.8396 Å if cations are ignored. Despite suggestions in other sources that NSAT might not be cubic (Steins *et al.*, 1997 ▸), we do not observe anything suggesting this from our lab XRD measurements or with TEM. For most applications as a substrate, this knowledge should be sufficient. Upon a more detailed examination it is apparent the structure is actually quite complex and is best described by a cubic *Fm*3*m* symmetry unit cell (*a* = 7.6856 Å) to account for the Al/Ta superstructure ordering along (111) planes (see Fig. 12 ▸). This superstructure exhibits extremely short-range order and poorly developed long-range order. It extends without disruption only over a few nm-sized domains. The high prevalence of resulting anti-phase domain walls is a likely reason for significant local disorder observed in IR and Raman spectroscopy. Qualitatively it was observed that the crystals are likely to be internally stressed as indirectly indicated by the insufficient quality of the SC-XRD measurements.

This assessment of the structure and disorder in NSAT crystals has implications for the use of NSAT as a substrate. We anticipate that NSAT will serve as a good substrate for perovskites and perovskite-related structures (*e.g.* Ruddlesden–Popper (Ruddlesden & Popper, 1957 ▸; Ruddlesden & Popper, 1958 ▸; Balz & Plieth, 1955 ▸), Dion–Jacobson (Dion *et al.*, 1981 ▸; Jacobson *et al.*, 1986 ▸; Gopalakrishnan *et al.*, 1987 ▸) or cuprate superconductor structures (Sunshine *et al.*, 1988 ▸; Watanbe *et al.*, 2003 ▸; Yu *et al.*, 2019 ▸) that are free of ordering on the *B*-sites. Since such films have no *B*-site order, they should be relatively insensitive to the ordering (and disorder to the ordering) present in NSAT. The utility of NSAT is that it provides a perovskite structural template at a lattice constant for which there are no other good perovskite substrates in the vicinity of it’s *a* = 3.843 Å unit-cell parameter. The closest commercial substrates are rhombohedral (and twinned) LaAlO_3_ to the lower pseudocubic (pc) unit-cell parameter side with *a*_pc_ = 3.791 Å and orthorhombic NdGaO_3_ to the higher lattice constant side with *a*_pc_ = 3.860 Å. Where NSAT might be problematic, however, is as a substrate for the growth of ordered double-perovskites (*e.g.* the room-temperature ferrimagnets Sr_2_FeMoO_6_ (Hauser *et al.*, 2011 ▸; Hauser *et al.*, 2012 ▸) and Sr_2_CrWO_6_ (Philipp *et al.*, 2003 ▸) as well as the ordered double-perovskite multiferroics like Bi_2_FeCrO_6_ (Baettig & Spaldin, 2005 ▸; Nechache *et al.*, 2009 ▸) and Bi_2_NiMnO_6_ (Azuma *et al.*, 2005 ▸), where the confusion of the *B*-site order in NSAT substrate and frequent anti-phase boundaries could lead to the lack of order in the resulting double-perovskite film grown upon the NSAT. Of course, there are currently no commercial double-perovskite substrates that are well ordered so this latter concern about NSAT is one for which there is no good current alternative. We thus expect NSAT to be a useful substrate for the growth of perovskite and perovskite-related films and strain engineering. Decreasing the residual strain in NSAT would likely improve its utility as a future substrate.

## Supplementary Material

Crystal structure: contains datablock(s) Fm3m_270625. DOI: 10.1107/S2052520626000703/bal5001sup1.cif

CCDC reference: 2529364

## Figures and Tables

**Table:** The site occupancies provided are the best mathematical fit, hence, the resulting formula is not entirely charge balanced.

Space group	*Pm* 3 *m*
*a* (Å)	3.841 (4)
*R*_int_ (%)	3.62
*R*1 (%)	5.40
*wR*_2_ (%)	16.71
GoF	1.294
Δρ_max_, Δρ_min_ (e Å^−3^)	3.6, −2.7

**Table:** 

Wyckoff site	Element	*x*, *y*, *z*	Occupancy
1*a*	Nd, Sr	0, 0, 0	0.36, 0.55
1*b*	Ta, Al	0.5, 0.5, 0.5	0.42, 0.57
3*c*	O	0, 0.5, 0	1

**Table:** 

Space group	*Fm* 3 *m*
*a*	7.7153 (2)
*R*_int_ (%)	6.19
*R*1 (%)	3.53
*wR*_2_ (%)	9.45
GoF	1.239
Δρ_max_, Δρ_min_ (e Å^−3^)	2.0, −2.4
Calculated chemical formula:	(Nd_0.426_, Sr_0.5740_)(Al_0.6010_, Ta_0.3990_)O_3_

**Table:** 

Wyckoff site	Element	*x*, *y*, *z*	Occupancy
4*a*	Al, Ta	0, 0, 0	0.875, 0.125
4*b*	Al, Ta	0.5, 0.5, 0.5	0.327, 0.673
8*c*	Nd, Sr	0.25, 0.25, 0.25	0.426, 0.574
24*e*	O	0, 0.5, 0	1

**Table 2:** IR-model parameters of the two detected transversal optical (TO) IR active phonon modes in NSAT Amplitude *S*, resonance frequency ω_TO_, broadening γ_TO_, and the dielectric limit *ɛ_∞_* [see equation (1)] are listed. Additionally, the second zeros (the first being at ω_TO_) of the real part of the dielectric function ɛ_1_ (where the resonance frequency of LO phonon modes would be expected) are also listed.

ɛ_∞_ = 4.0	*S*	ω_TO_ (cm^−1^)	γ_TO_ (cm^−1^)	Second zero (cm^−1^)
T_1u_ (1)	0.6	662	43	774
T_1u_ (2)	3.2	399	48	570

**Table 3:** Comparison of the positions of the Raman bands (cm^−1^) for similar Sr(Al_0.5_Ta_0.5_)O_3_ (SAT) and La*_x_*Sr_1–*x*_Al_1–0.5*x*_Ta_0.5*x*_O_3_ (LSAT) materials and NSAT measured here

	A_1g_	T_2g_	E_g_
LSAT (Runka *et al.*, 2004 ▸)	875	467	591
SAT (Tao *et al.*, 1996 ▸)	872	467	578
NSAT	881	474	556

The UV–vis absorption spectrum measured is consistent with the one reported previously (Pawlak *et al.*, 2005 ▸), however, the transmittance values are slightly higher.
